# Single-Cell Heterogeneity in Snake Venom Expression Is Hardwired by Co-Option of Regulators from Progressively Activated Pathways

**DOI:** 10.1093/gbe/evad109

**Published:** 2023-06-13

**Authors:** Aundrea K Westfall, Siddharth S Gopalan, Blair W Perry, Richard H Adams, Anthony J Saviola, Stephen P Mackessy, Todd A Castoe

**Affiliations:** Department of Biology, The University of Texas Arlington, Texas, USA; Department of Biology, The University of Texas Arlington, Texas, USA; Department of Biology, The University of Texas Arlington, Texas, USA; School of Biological Sciences, Washington State University, Pullman, Washington, USA; Department of Entomology and Plant Pathology, University of Arkansas, Fayetteville, USA; Department of Biochemistry and Molecular Genetics, University of Colorado Denver, Aurora, USA; School of Biological Sciences, University of Northern Colorado, Greeley, USA; Department of Biology, The University of Texas Arlington, Texas, USA

**Keywords:** ERK, unfolded protein response, gene regulatory networks, chromatin, single-cell RNAseq

## Abstract

The ubiquitous cellular heterogeneity underlying many organism-level phenotypes raises questions about what factors drive this heterogeneity and how these complex heterogeneous systems evolve. Here, we use single-cell expression data from a Prairie rattlesnake (*Crotalus viridis*) venom gland to evaluate hypotheses for signaling networks underlying snake venom regulation and the degree to which different venom gene families have evolutionarily recruited distinct regulatory architectures. Our findings suggest that snake venom regulatory systems have evolutionarily co-opted *trans*-regulatory factors from extracellular signal-regulated kinase and unfolded protein response pathways that specifically coordinate expression of distinct venom toxins in a phased sequence across a single population of secretory cells. This pattern of co-option results in extensive cell-to-cell variation in venom gene expression, even between tandemly duplicated paralogs, suggesting this regulatory architecture has evolved to circumvent cellular constraints. While the exact nature of such constraints remains an open question, we propose that such regulatory heterogeneity may circumvent steric constraints on chromatin, cellular physiological constraints (e.g., endoplasmic reticulum stress or negative protein–protein interactions), or a combination of these. Regardless of the precise nature of these constraints, this example suggests that, in some cases, dynamic cellular constraints may impose previously unappreciated secondary constraints on the evolution of gene regulatory networks that favors heterogeneous expression.

**Significance**

Cellular heterogeneity is an important component of organismal phenotypes, which raises questions about how gene regulatory networks that produce such heterogeneity evolve. Here, we leverage single-nucleus RNA sequencing of a rattlesnake venom gland to explore the relationships between cellular heterogeneity and gene regulatory networks underlying venom expression. We find that venom expression heterogeneity correlates with activation of pathways proposed to regulate venom and that venom genes have specifically recruited transcription factors from regulatory pathways that exhibit inherently heterogeneous activity via their phased activation. Our inferences highlight an insightful evolutionary solution for how heterogeneous expression may evolve through the evolutionary co-option of transcription factors from existing regulatory cascades that exhibit phased responses.

## Introduction

Understanding how new complex physiological traits arise via the evolution of gene regulatory networks (GRNs), and how cellular variation in GRNs manifests in organismal phenotypes, is fundamental to understanding the processes that generate phenotypic diversity. Cells that comprise complex tissues often express different sets of genes due to functional differentiation between cell types; such cellular heterogeneity is found ubiquitously across many tissue types (Heindl et al. 2015; Ben-Moshe and Itzkovitz 2019). Cellular heterogeneity can also arise among otherwise identical classes of cells because of multiple systems of messenger RNA (mRNA) expression that support the same outcomes (Eberwine and Kim 2015), such as modular combinations of *trans*-acting factors that activate expression or intrinsic heterogeneity in response to stress (Gasch et al. 2017; Konstantinides et al. 2018). Complex eukaryotic phenotypes are often a result of cellular heterogeneity (Altschuler and Wu 2010; Paszek et al. 2010; Ackermann 2015; Márquez-Zacarías et al. 2021), and new spatial and single-cell approaches are enhancing our appreciation of its importance and how it contributes to organism-level phenotypes (Papalexi and Satija 2018; Stuart et al. 2019; Taylor et al. 2021). Such high-resolution studies have highlighted the roles of varying developmental trajectories (Bendall et al. 2014; Farrell et al. 2018), regulatory pathway activation (Yang et al. 2017), and differential sensitivities to stimuli that underlie patterns of cellular variation within tissues (Adelaja et al. 2021), even among venom-producing tissues (Columbus-Shenkar et al. 2018; Sachkova et al. 2019; Verdes et al. 2022). The observation of widespread cellular heterogeneity raises broad questions about what gene regulatory mechanisms may generate such heterogeneity, how these mechanisms may evolve, and what ultimate cellular constraints may shape these regulatory architectures.

Snake venom systems, including the specialized glands that produce venom, have emerged as models for studying the evolution of novel complex traits and the evolutionary co-option of genes and regulatory systems underlying them (Mackessy and Baxter 2006; Perry et al. 2020; Post et al. 2020; Zancolli and Casewell 2020; Puschhof et al. 2021; Hempel et al. 2022; Kazandjian et al. 2022). Snake venom systems are a particularly valuable model for investigating the origins and evolution of regulatory networks due to the moderate number of distinct venom gene families recruited for venom and the direct relationships between venom gene expression, organismal phenotype, and fitness (Casewell et al. 2012, 2013; Heindl et al. 2015; Rokyta et al. 2015; Holding et al. 2016; Zancolli and Casewell 2020; Perry et al. 2022). The evolution of snake venom entailed the recruitment, and often subsequent expansion via tandem duplication, of over 20 gene families that contribute toxic proteins to snake venoms (Wong and Belov 2012; Schield et al. 2019; Suryamohan et al. 2020; Zancolli and Casewell 2020). This process necessitated the rewiring of regulatory networks to precisely control the expression of these toxic proteins in specialized secretory glands (Barua and Mikheyev 2021; Perry et al. 2022). Recent tissue-level functional genomic studies suggest that multiple snake venom gene families have been evolutionarily rewired to be regulated by a large suite of transcription factors (TFs) controlled by two conserved regulatory pathways: the extracellular signal-regulated kinase (ERK) and the unfolded protein response (UPR) (Barua and Mikheyev 2021; Perry et al. 2022). These studies also proposed that the integration of these two pathways leads to a positive feedback mechanism that further upregulates venom expression as the UPR pathway is activated by cellular and endoplasmic reticulum (ER) stress following the initial stages of venom production initiated by ERK signaling (Perry et al. 2022). These predictions for the roles of higher-level signaling pathways and of specific TFs in regulating snake venom gene families have not, however, been further explored in other experimental contexts.

Spatial heterogeneity of venom toxin expression within snake venom glands has also recently been demonstrated using proteomic imaging, which highlighted the discrete localization of toxic peptide expression across the tissue (Hempel et al. 2022). Single-cell RNA sequencing (scRNAseq) resolution of this phenomenon has been limited to a single study focused on venom gland organoids that compared scRNAseq data from venom gland organoids derived from an elapid snake species with data from venom gland tissues from the same species, primarily for the purpose of demonstrating that organoids and glands recapitulate similar patterns of heterogeneity (Post et al. 2020). These prior studies used species that lacked reference genomes, which prevented interpretation of these data in a broader context of genomic architecture or regulatory pathways. These prior studies also hypothesized that observed heterogeneity in snake venom glands might be developmentally programmed (Post et al. 2020; Kazandjian et al. 2022) and might be a consequence of unidentified physiological constraints that require partitioning of venom toxin expression across secretory cells (Kazandjian et al. 2022). However, no prior studies have characterized venom gland cellular heterogeneity in a systematic way or linked these patterns of venom expression heterogeneity to the GRNs that may direct this heterogeneity. We hypothesize that glandular depletion activates GRNs within secretory cells that direct venom gene expression in specific stages that manifest as venom gene expression heterogeneity across venom secretory cells. In effect, rather than partitioning venom gene expression across a gland via regulation by distinct GRNs, expression of distinct venom genes occurs at different stages of activation of a shared GRN.

To better understand the causes and consequences of cellular heterogeneity in the snake venom gland and test our hypotheses for an explanation of venom expression heterogeneity, we generated single-nucleus RNA sequencing (snRNAseq) from the venom gland of the prairie rattlesnake (*Crotalus viridis*). This species is associated with extensive genomic resources, including a chromosome-level reference genome (Schield et al. 2019) and tissue-level functional genomic inferences of venom gland physiology, venom variation, and GRNs underlying venom regulation (Mackessy 1991; Schield et al. 2019; Perry et al. 2020, 2022; Zancolli et al. 2022), which provide valuable context for the integration of inferences from single-cell approaches. Using snRNAseq data from the venom gland of this species, we investigate patterns of variation and covariation among venom genes and genes that encode *trans*-acting factors to infer the mechanisms that explain cellular heterogeneity in venom expression. We also evaluate evidence for ERK and UPR activation in relation to venom expression, infer de novo GRNs that differ between venom gland secretory cell populations, and compare these inferences to recent hypotheses for GRNs that regulate venom in snakes. Finally, we integrate our snRNAseq and prior tissue-level functional genomic data to evaluate the hypothesis that snake venom systems have systematically recruited suites of TFs that inherently generate cellular heterogeneity in venom gene expression. Our findings suggest that cellular heterogeneity in venom gene expression is a mechanistic consequence of the evolutionary recruitment of distinct suites of TFs that are linked to phased activation of ERK and UPR pathways in secretory cells.

## Results

### Patterns of Tissue-Level and Single-Cell Expression in Venom Glands

**Fig. 1.**
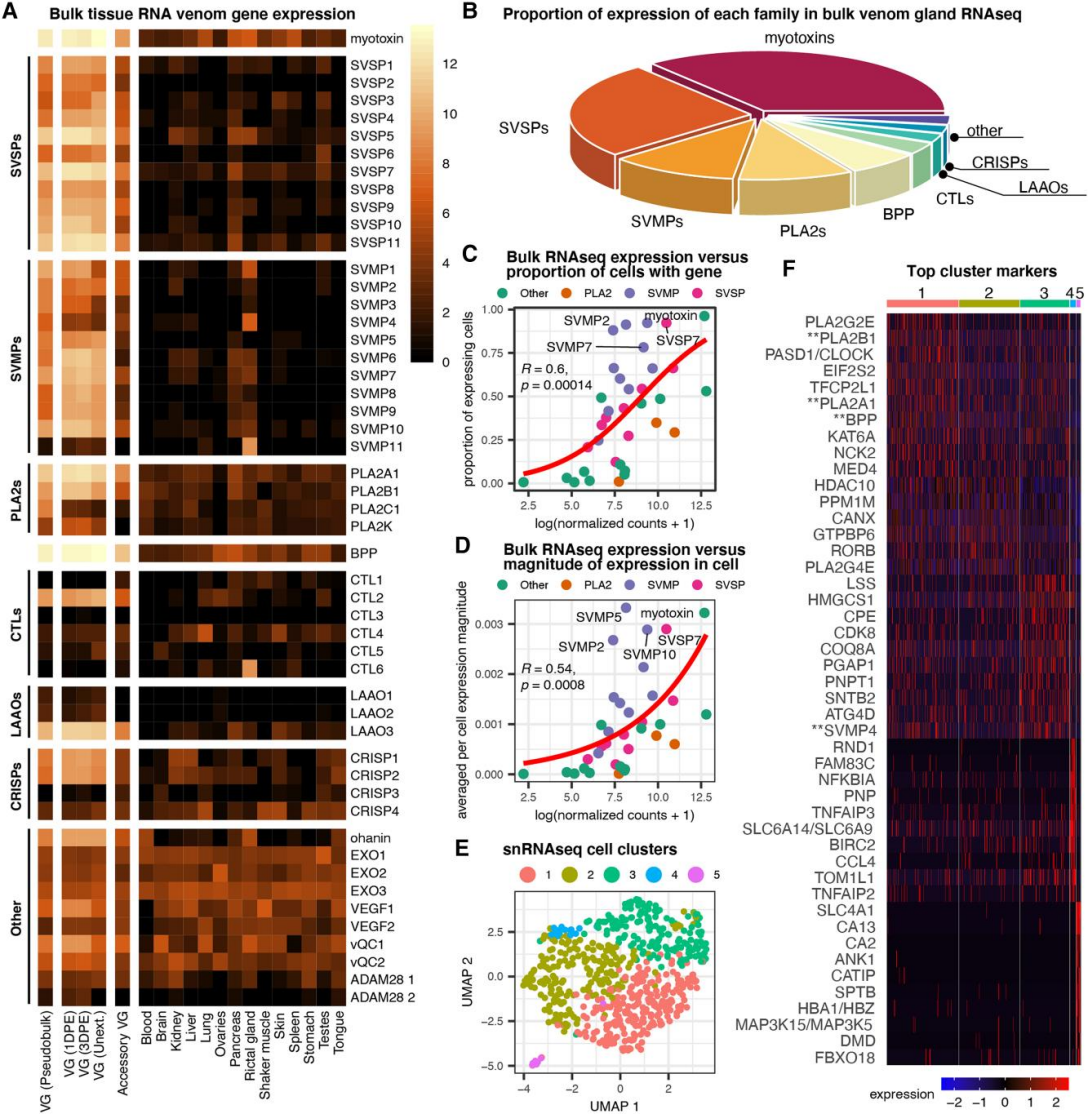
Patterns of tissue-level and single-cell expression in venom glands. (*A*) Heatmap of venom gene expression in both venom gland and body tissue RNAseq. (*B*) Proportion of venom proteome comprised by each venom gene family (redrawn from Saviola et al. 2015). (*C*) Bulk tissue expression is correlated with the proportion of cells expressing the toxin. (*D*) Tissue-level expression is correlated with the average magnitude of expression from single cells (i.e., tissue-level expression is driven by a combination of a greater number of expressing cells as well as higher per-cell expression). (*E*) Naïve clustering of cells from single-nucleus RNAseq of a venom gland. (*F*) Top 10 marker genes for each cluster. Double asterisks (**) denote venom genes. BPP, bradykinin potentiating peptide; CRISP, cysteine-rich secretory protein; CTL, C-type lectin; DPE, days postextraction; LAAO, L-amino oxidase; PLA_2_, phospholipase A_2_; RVG, right venom gland; SVMP, snake venom metalloprotease; SVSP, snake venom serine protease; Unext., unextracted.

To confirm that our snRNAseq data were representative of whole venom gland gene and protein expression, we compared our pseudobulk (merged snRNAseq counts across cells) data to previously published tissue-level mRNAseq from multiple tissues (including venom gland) and venom gland proteomic data from *C. viridis* (Saviola et al. 2015; Schield et al. 2019) (fig. 1*A* and *B*). This confirms high similarity between overall venom gene expression from our snRNAseq data to multiple biological replicates of tissue-level mRNAseq and venom proteomic composition. Direct comparisons of venom gene expression between tissue-level mRNAseq and snRNAseq data also demonstrate that both the proportion of cells expressing a given venom toxin and the relative magnitude of expression per cell correlate with tissue-level mRNAseq toxin expression inferences (fig. 1*C* and *D*).

Our snRNAseq experiment yielded 619 nuclei with 761,564 mean reads per cell and 506 mean genes per cell. Overall gene expression variation grouped cells into five naïve clusters, including three weakly differentiated clusters (clusters 1–3) of venom-secreting cells and two smaller clusters (clusters 4 and 5) comprised of hematopoietic cells and other nonvenom-secreting cells (fig. 1*E*). Across these five clusters, we identified marker genes that most highly differentiate each cluster (fig. 1*F*). For cluster 1, the top markers include multiple venom genes (*PLA2B1*, *PLA2A1*, and *BPP*), *PLA2G4E*, a nonvenom PLA_2_ family gene, and *RORB*, which has been previously implicated in venom regulation (Perry et al. 2022). Among cluster 3 top markers are the venom gene *SVMP4* and *CDK8*, a TF downstream of EGFR signaling, which has also been implicated in venom gene regulation (Casewell et al. 2013). In contrast to secretory cell clusters (1–3), clusters 4 and 5 contain fewer cells and encompass hematopoietic cells. Leukocytes in cluster 4 express the lymphocyte marker *PNP* (Maeda et al. 1981). Cluster 5 includes erythrocytes that express a red blood cell membrane component (*ANK1*) and a hemoglobin ortholog (*HBD*). Additionally, we explored the presence of markers identified in previous venom gland analyses (Post et al. 2020) and identified expression of *EPCAM* throughout the secretory epithelial cells (clusters 1–3) and *HEMGN* in hematopoietic cells (supplementary fig. S1, Supplementary Material online), confirming our cluster cell-type assignments.

### Cell Clustering Based on Venom Gene Expression

**Fig. 2.**
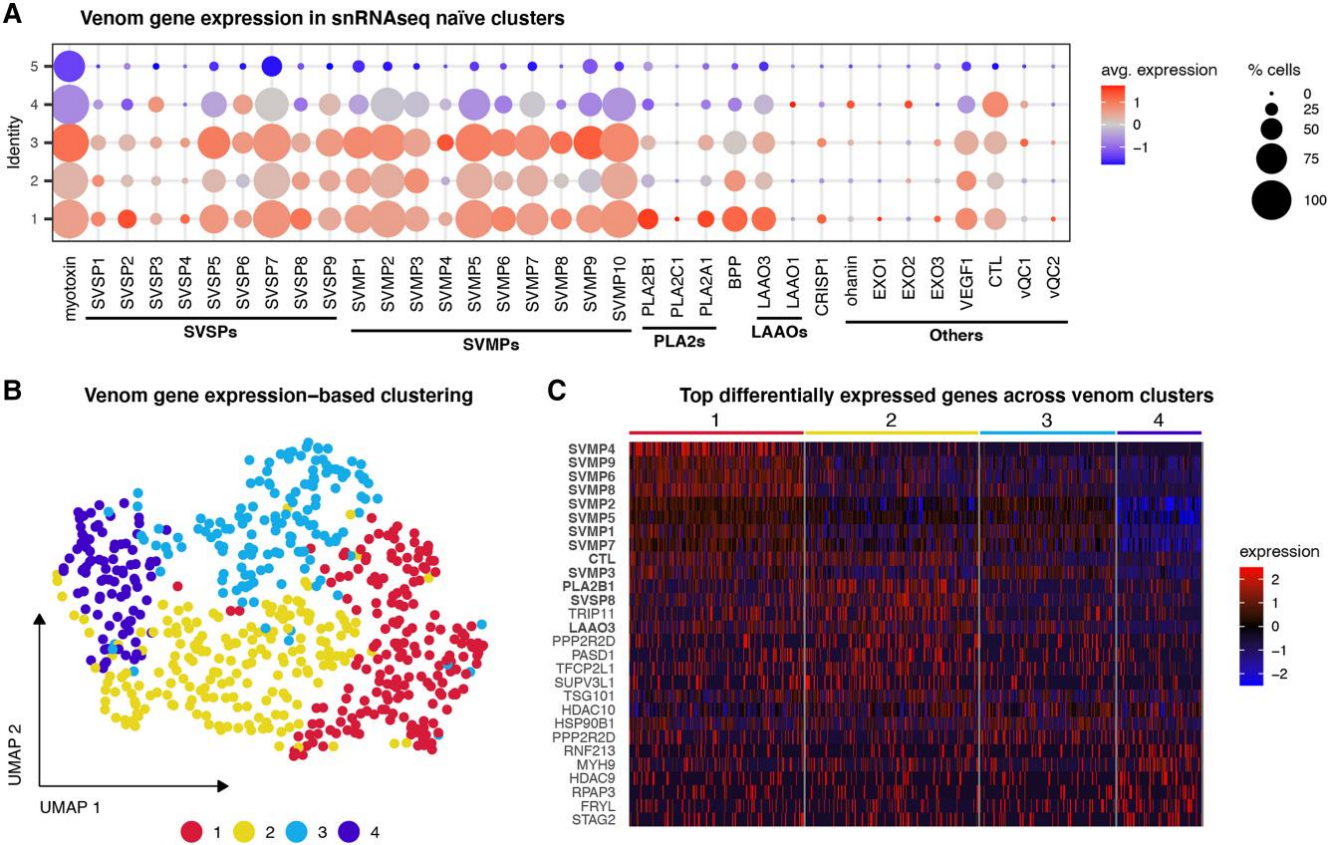
Cell clustering based on venom gene expression. (*A*) Expression by Seurat-based cluster identity of each venom gene detected in our snRNAseq data shows low variation for each venom gene in each cluster. (*B*) Uniform manifold approximation and projection (UMAP) cell clusters generated based on the expression of venom genes. (*C*) Heatmap of top 10 differentially expressed genes of each venom gene-based cluster. Venom genes are shown in bold. Some genes are markers for multiple clusters.

Because venom gene expression does not display notable variation across the five naïve clusters based on expression of all genes (fig. 2*A*), we reclustered cells based only on expression variation of venom genes, which identified four cell clusters (fig. 2*B*). Cell clusters inferred from all genes versus clusters based on venom genes only have minimal overlap (supplementary fig. S2, Supplementary Material online), indicating that venom gene expression is heterogeneous within secretory cell populations. Based on clustering using venom gene expression only, cluster 1 is primarily defined by high snake venom metalloprotease (SVMP) gene expression in contrast to other clusters, which have high expression of a smaller number of SVMPs and *PLA2B1*, *LAAO3*, and *CTL* (fig. 2*C*). Other major cluster markers include histone deacetylases (*HDAC9* and *HDAC10*), major TFs (*PASD1* and *TFCP2L1*), transport-associated proteins (*TRIP11* and *TSG101*), molecular chaperones (*HSP90B1* and *RPAP3*), phosphatases (*PPP2R2D*), and cytoskeletal proteins (*MYH9*). Further subclustering of these groups identified cell clusters with relatively high levels of expression of specific venom genes, including multiple SVMP paralogs, *PLA2A1*, *PLA2B1*, and myotoxin (supplementary fig. S3, Supplementary Material online).

### Gene Structure, Chromatin, and Expression Variation within and across Tandemly Duplicated Venom Gene Families

Cellular constraints that require distinct venom toxins to be expressed in distinct subsets of secretory cells have been hypothesized to drive expression heterogeneity within the venom gland (Kazandjian et al. 2022). To investigate this, we calculated pairwise correlations of gene expression across cells for paralogs within and among venom gene families. SVMP and SVMP paralogs—which are also among the highest expressed venom genes across all cells (fig. 2*A*)—show the highest correlations of within-family paralog expression across cells (supplementary fig. S4, Supplementary Material online). In contrast, the expression of the two major PLA_2_ paralogs, *PLA2A1* and *PLA2B1*, are not significantly correlated (false discovery rate [FDR] = 0.94). A key distinction between these families is their size and intergenic distances within these gene arrays, with the PLA_2_ cluster containing four paralogs within a relatively condensed cluster (total array length: ∼30 kb), compared with the larger SVMP (11 paralogs; ∼600 kb) and SVSP (9 paralogs; ∼500 kb) clusters. Other venom genes tend to have weakly positive or negative correlations of expression across and within families except for some highly expressed venom genes (e.g., myotoxin, *LAAO3*, and *CTL*). However, these correlations lack statistical significance (i.e., FDR ≥ 0.05), indicating that venom gene expression poorly correlated among gene families across secretory cells.

By combining single-nucleus coexpression information with prior predictions of three-dimensional chromatin structure and loop conformation around these gene arrays (Perry et al. 2022), we assessed whether venom gene expression heterogeneity could be explained by characteristics of gene or chromatin structure, including intergenic spacing and the predicted binding of the insulator *CTCF*. We find evidence that small intergenic distance has the potential to play a role in generating constraints that lead to expression heterogeneity. Related to the broader hypothesis of constraints underlying patterns of heterogeneity, we also find that the PLA_2_ inhibitor, a peptide inhibitor of PLA_2_ venom toxins previously found to be a cluster marker in venom gland organoids (Post et al. 2020), is rarely coexpressed with venom PLA_2_s (supplementary fig. S5, Supplementary Material online).

### GRN Activity Differentiates Subpopulations of Venom Secretory Cells

**Fig. 3.**
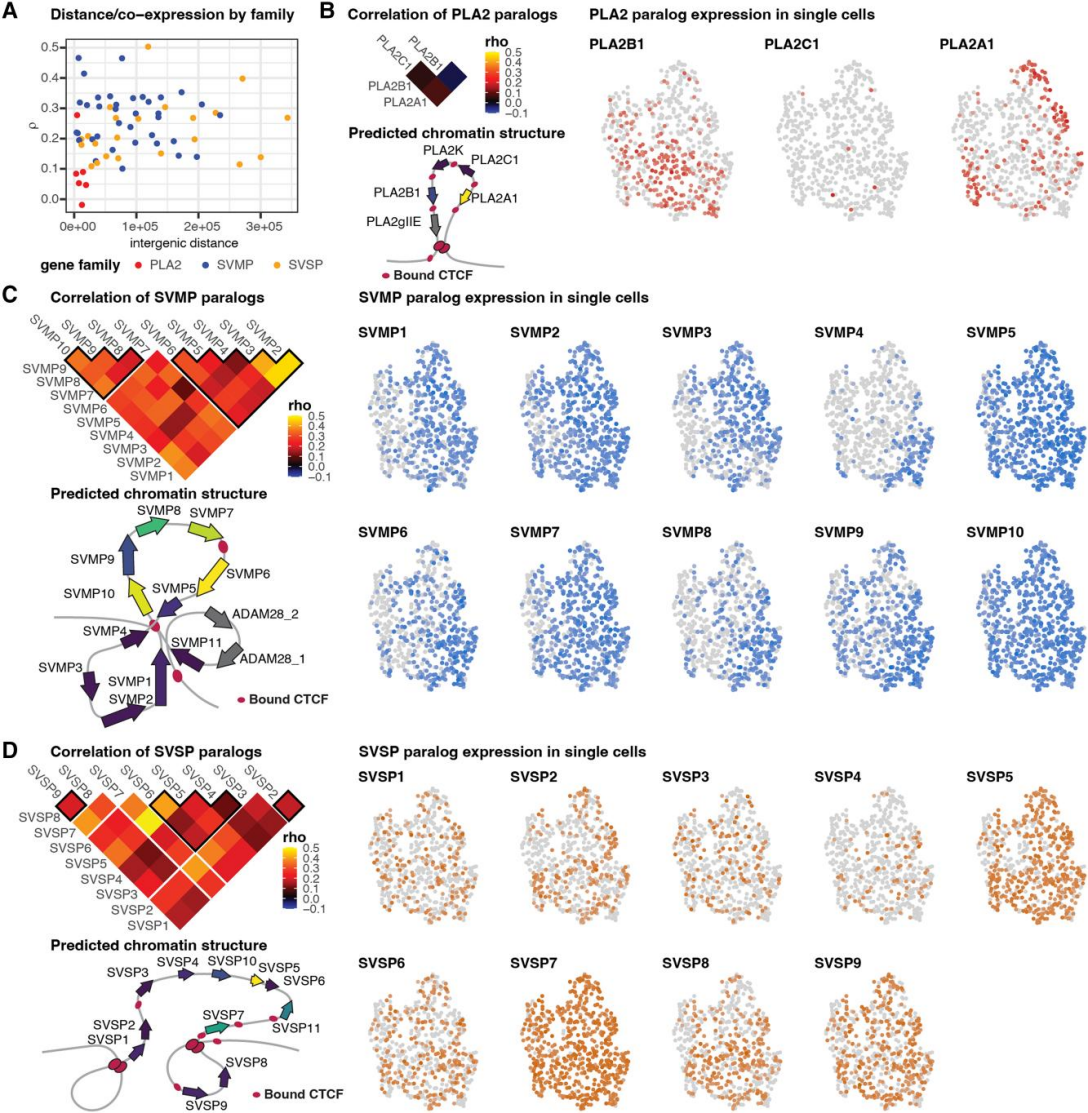
Gene structure, chromatin, and expression variation within and across tandemly-duplicated venom gene families. (*A*) A plot of gene–gene correlation coefficients and intergenic distances between genes indicates the potential role of steric constraints on coexpression in PLA_2_s. Additional panels display family-wise paralog correlation matrices subset from (*A*) (gaps in the matrix denote predicted locations of bound CTCF and black outlines indicate genes grouped within CTCF boundaries), gene expression plotted on the venom-gene–based UMAP from (*B*) and the predicted chromatin structure and gene expression of each array (redrawn from Perry et al. 2022) for PLA2s (*B*), SVMPs (*C*), and SVSPs (*D*). Note that PLA2K, SVSP10, and SVSP11 were not detected in our single-cell expression data.

To investigate GRNs that may underlie heterogeneity in venom expression across secretory cells, we inferred regulons (i.e., high-level TFs and their direct interactors) underlying cell variation using SCENIC (Aibar et al. 2017). We identified 96 regulons that show differential activity across the venom expression-variable cell clusters (supplementary fig. S6, Supplementary Material online). We then measured the relationship between regulon activity and venom gene expression and categorized the top-level TF of each regulon based on their relationships to the ERK and UPR pathways, their roles in chromatin modification, and whether these TFs have been previously implicated in regulating venom genes (fig. 3*A* and supplementary fig. S6 and table S1, Supplementary Material online). We find that the per-cell activity of the *CREB3L2*, *SMARCA4*, and *SREBF1* networks are most strongly correlated with the expression of the highly expressed SVSP and SVMP gene families as well as myotoxin (fig. 3*A*). Gene ontology (GO) term and Kyoto Encyclopedia of Genes and Genomes (KEGG) pathway enrichment of the downstream targets of *CREB3L2*, *SMARCA4*, and *SREBF1* are frequently shared, and both *CREB3L2* and *SMARCA4* target protein processing and localization to the endoplasmic reticulum, implicating these TFs in a coordinated high-level response to venom depletion (supplementary fig. S6, Supplementary Material online). Additionally, multiple regulons are associated with TFs involved in the UPR (e.g., *ATF6* and *CREB3L2*), interact with *ERK* (e.g., *BRCA1*, *ELK1*, and *ETS2*), or are involved in histone modifications and chromatin structure (e.g., *EP300*, *CTCF*, *HCFC1*, *HDAC2*, *HDAC6*, and *KDM5B*). These analyses also independently recovered multiple regulons previously proposed as key regulators of venom gene expression based on independent lines of evidence in prior tissue-level experiments (Perry et al. 2022), including *MEIS1*, *XBP1*, *CEBPZ*, and *ATF4*. Other regulon TFs identified here for the first time may also play roles in venom gland physiology (fig. 3*A*, “*Novel inference”).

To understand how the regulatory networks underlying the physiological specialization of the venom secretory gland and networks hypothesized to specifically regulate venom gene expression (Perry et al. 2022) relate to each other, we inferred the interaction network among regulons. We find that 88 regulons form a single interaction network, in which the highest-degree (most highly connected) node is *EP300*, a hub histone acetyltransferase (Eckner et al. 1994), suggesting a conserved role across oral and salivary secretory glands. Several of the top interacting TFs are classified as chromatin modifiers and as factors related to ERK/UPR signaling, further highlighting the pervasive links between glandular regulatory networks and those associated specifically with venom regulation. Together, this model of venom gland regulatory architecture highlights the degree to which venom GRNs were co-opted from and directly integrated within the regulatory structure and underlying physiology of the venom secretory gland.

To complement our top-down inferences of regulatory networks, we constructed a naïve de novo interaction network incorporating nonvenom genes with strong correlations with venom gene expression (protein-protein interaction enrichment *P*-value < 0.05; supplementary fig. S8, Supplementary Material online). A subset of genes in the network are overrepresented in GO and KEGG pathway terms, including protein processing in the endoplasmic reticulum (hsa04141), antigen processing and presentation (GO: 0019884), and nuclear transport (GO: 0051169). Additional annotations of interest for genes in this network include associations with chromatin remodeling, mRNA decay, and a pioneer TF implicated previously in regulating snake venom genes (*GRHL1*) (Perry et al. 2022).

### Cellular Venom Gene Expression Variation Is Associated with ERK and UPR Signaling Pathways

**Fig. 4.**
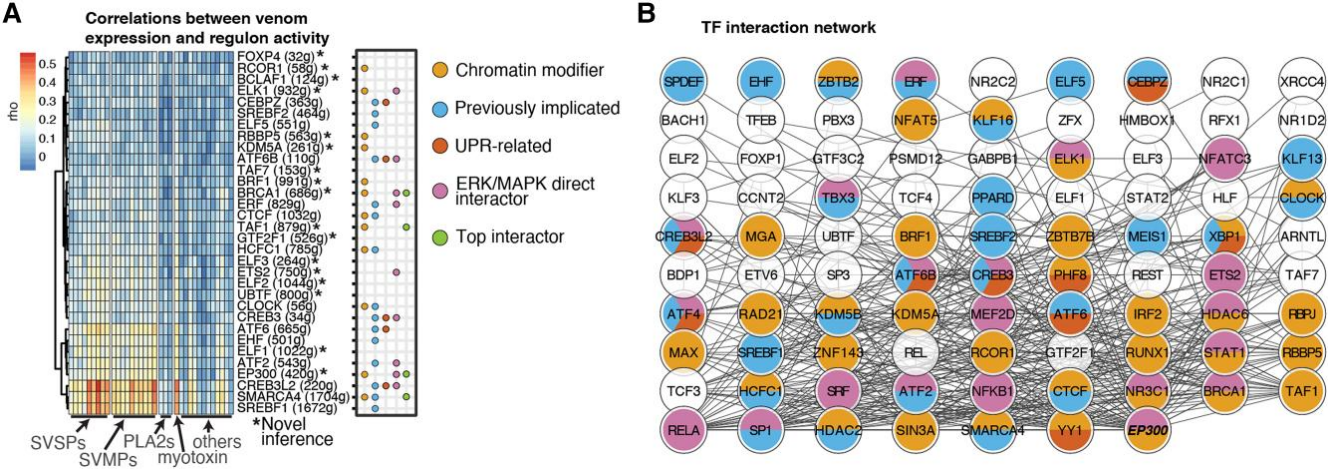
GRN activity differentiates subpopulations of venom secretory cells. (*A*) A reduced Pearson's rho matrix of regulon activity measured by AUCell (Aibar et al. 2017) and venom gene expression across single cells. The number of genes in each regulon is shown in brackets. The first four categorization columns represent functional categories and TFs previously implicated in venom gene regulation, and the fifth represents the top 10 TFs sorted by degree in their interaction network. (*B*) The degree-sorted interaction network of high-level TFs from STRINGDB (Szklarczyk et al. 2021); *EP300* is the highest degree node.

Prior studies inferred that the UPR and ERK signaling pathways were evolutionarily co-opted to regulate snake venom genes and may operate in a positive feedback mechanism that further upregulates venom gene expression upon UPR activation by endoplasmic reticulum stress (Perry et al. 2022). To investigate this hypothesis, we tested for relationships between ERK and UPR pathway activation and venom expression. We find that single-cell gene set enrichment of genes involved in both ERK and UPR is significantly correlated with enrichment of venom genes (fig. 4*A* and *B*). We also find that enrichment scores for ERK and UPR are strongly correlated across cells, supporting the hypothesis that the integrated stimulation of both pathways activates venom gene expression (fig. 4*C*) (Perry et al. 2022).

### Regulatory Element Binding and Gene Coexpression Support ERK and UPR Function in Venom Glands

To further explore how ERK and UPR activation may relate to the heterogeneous expression of specific venom genes, we calculated expression of *k*-means clustered cells along the continuums of ERK–UPR enrichment (fig. 4*D*). We find that expression of distinct sets of venom genes, including paralogs, varies with the joint state of ERK and UPR activation within cells (fig. 4*E*). These findings raise the possibility that venom genes may demonstrate cellular expression heterogeneity based on having recruited specific TFs involved in ERK and UPR activation that have ordered phases of activation in secretory cells.

**Fig. 5.**
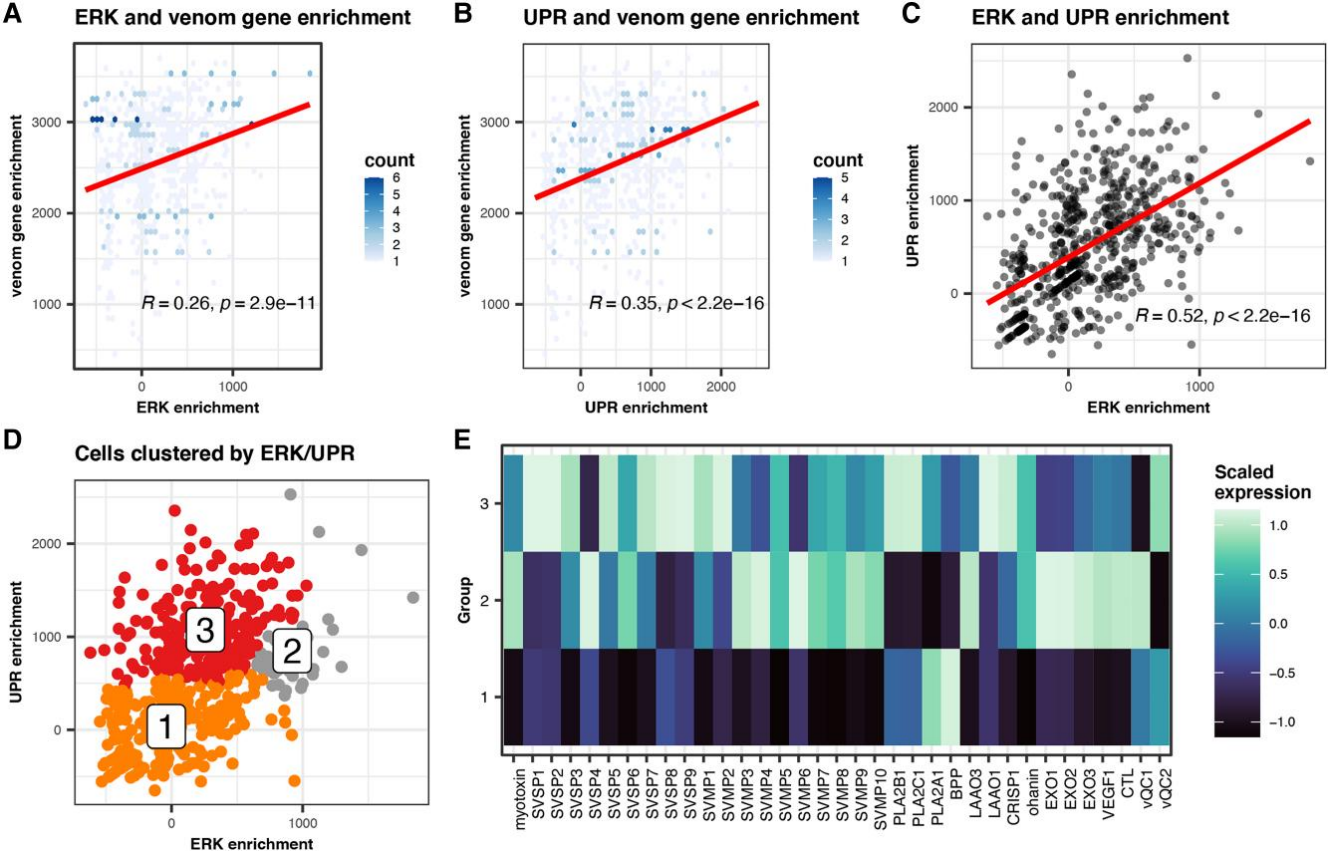
Cellular venom gene expression variation is associated with ERK and UPR signaling pathways. (*A*) ERK and (*B*) UPR pathway gene enrichment correlated with venom gene enrichment in each cell. Hexes are colored by density of cells. (*C*) Significant correlation of ERK and UPR pathway enrichment across venom gland cells. (*D*) Cells cluster by joint ERK and UPR enrichment. (*E*) Specific venom genes respond in expression to different phases of the ERK–UPR continuum. Rows labeled 1–3 represent the expression of the same groups of cells labeled in (*D*).

To test the hypothesis that TFs recruited to regulate venom are associated with phased ERK and UPR pathway activation, we integrated data from 1) prior predictions of TFs bound at *cis*-regulatory elements (CREs) of venom genes in *C. viridis* (Perry et al. 2022), 2) annotations of the roles of TFs in ERK and UPR, and 3) single-cell network adjacency weights calculated using GENIE3 (Huynh-Thu et al. 2010), an indication of importance between the expression of a regulator and its target, providing an estimate of the strength of the regulatory relationship between two genes within the network (fig. 5*A*). These comparisons indicate that different venom gene families (and paralogs within families) are associated with expression of distinct UPR/ERK TFs (adjacency scores; fig. 5*A*) and have CREs that bind TFs associated with distinct phases of the UPR and ERK pathways (ATACseq [Assay for Transposase-Accessible Chromatin sequencing] footprints; fig. 5*A*). This pattern is consistent with the hypothesis that venom gene expression heterogeneity is driven by phased activation of distinct UPR- and ERK-related TFs that regulate venom.

**Fig. 6.**
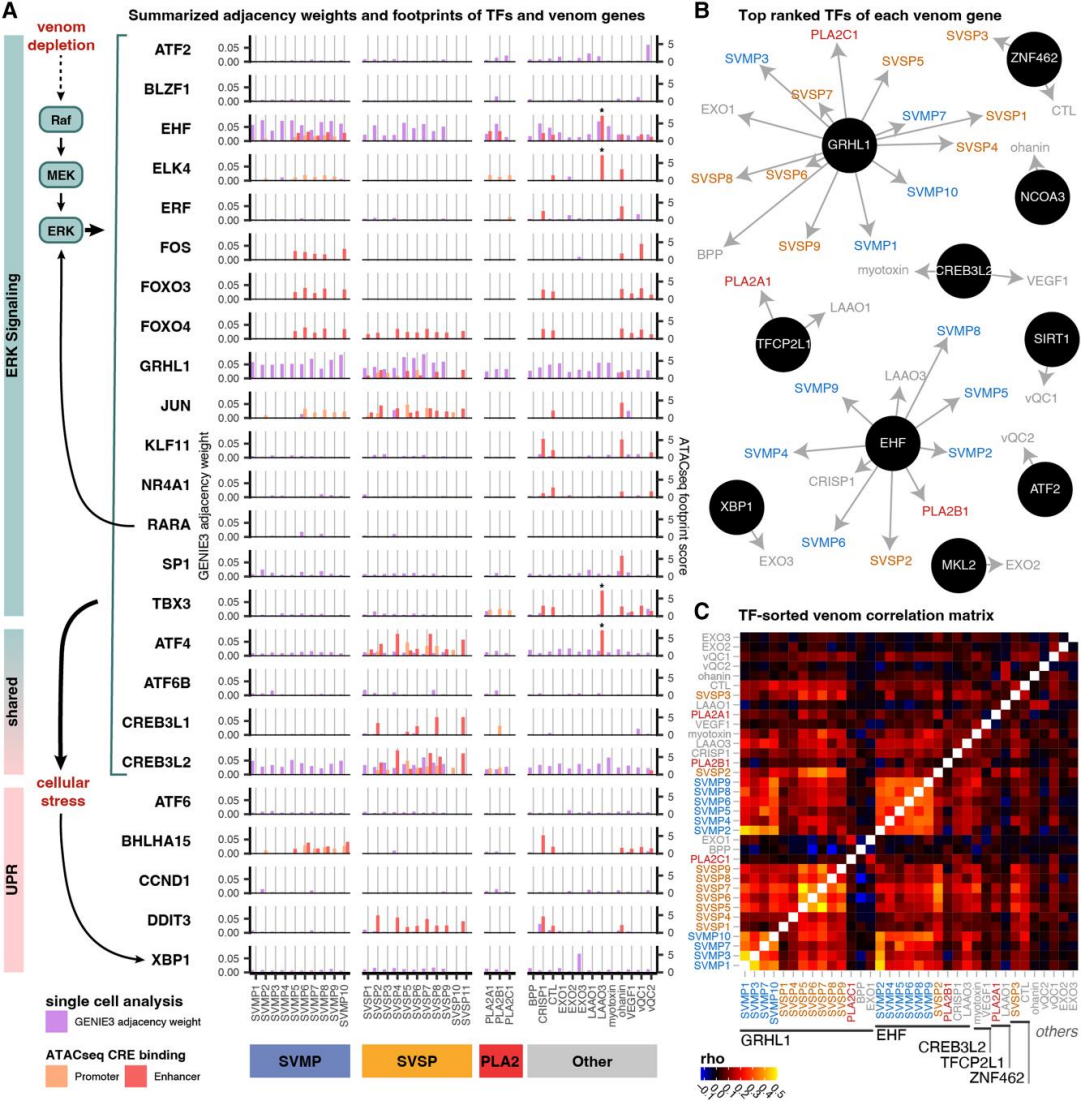
Regulatory element binding and gene coexpression support ERK and UPR function in venom glands. (*A*) Adjacency weights from GENIE3 from single-nucleus data (left axis) and footprint scores in promoters and enhancers from tissue-level ATACseq data (right axis) displayed for each venom gene relative to candidate UPR and ERK signaling TFs. A broad timeline of major pathway steps is depicted to the left. Asterisks (*) on LAAO3 enhancer weights indicate extreme values that exceed the depicted scale. (*B*) Directed network of TFs with the highest predicted GENIE3 importance score for each venom gene paralog. Arrows point from the TF to the venom gene predicted to be regulated. (*C*) Coexpression heatmap of venom genes, sorted by shared high-ranked TFs.

Our computational analyses integrating relationships between TF expression and venom gene expression predict that the expression of different venom genes is best explained by distinct TFs, implicating these predictive TFs as regulators of these venom genes. For example, each PLA_2_ paralog is best predicted by the expression of a different TF (fig. 5*B*), indicating that differential TF co-option underlies patterns of venom expression heterogeneity across cells (e.g., fig. 6*B*). These computational inferences also suggest that additional candidate TFs outside of the canonical ERK and UPR signaling cascades contribute to the cell-specific patterns of expression among venom genes (supplementary fig. S9, Supplementary Material online), likely via other interactions with *EHF*, *GRHL1*, and *CREB3L2*. We also find that expression of venom genes across cells is more highly correlated among venom paralogs that share CRE-TFs (fig. 5*C*), consistent with sets of TFs directing expression of distinct venom gene expression across cells. Together, our results support that variation in expression of venom genes across cells is inherently linked to, and directly regulated by, TFs co-opted from different nodes of ERK and UPR pathways that reflect different underlying phases of the activation of these pathways across secretory cells. Importantly, our results suggest that distinct gene families, as well as multiple distinct tandemly duplicated paralogs within gene families, appear to be regulated by distinct sets of TFs that are associated with distinct phases of the activation of both ERK and UPR pathways.

## Discussion

Our findings provide new evidence that links the proximal mechanisms that underlie snake venom gland heterogeneity to the evolutionary strategies by which venom gene regulation has co-opted regulatory networks. We demonstrate that the activity of both ERK and UPR signaling pathways is positively correlated with the expression of venom genes across venom gland secretory cells, further supporting the presence of a positive feedback relationship between these two pathways (Perry et al. 2022). Moreover, the expression of venom genes in each cell is related to the degree of activity of both ERK and UPR, which underlies a continuum of variation across secretory cells in response to venom depletion. Our findings suggest that overall cellular heterogeneity, including venom expression heterogeneity, reflects a spectrum of venom secretory cells at different phases of activation of these two pathways poststimulation—an axis over which venom gene expression changes immensely (Currier et al. 2012). Recent advances in snake venom gland organoid systems (Post et al. 2020; Puschhof et al. 2021) may eventually enable the testing of this prediction, by experimentally stimulating or inhibiting these two pathways in vitro, for example. This has important ramifications for understanding how snake venom systems have co-opted existing *trans*-regulatory cascades to exploit their heterogeneous, phased activation, orchestrating the heterogeneous production of venom proteins across cells in the process. Indeed, prior studies that observed heterogeneity in snake venom organoids have suggested that cellular heterogeneity may be an inherent “developmental” property of snake venom glands (Kazandjian et al. 2022); our results suggest this inherent property is driven by the dynamic variation in ERK and UPR signaling across secretory cells that venom genes have evolutionarily recruited.

Recent studies have hypothesized that TFs involved in the ERK and UPR signaling pathways have been co-opted to regulate snake venom (Barua and Mikheyev 2021; Perry et al. 2022). Our findings build on these prior inferences and highlight substantial heterogeneity in GRN activity across venom-secreting cells, much of which is associated with the ERK and UPR signaling pathways, as well as transcriptional regulation and chromatin remodeling. We identified several candidate genes that appear to be critical regulators of venom gland physiology, such as *EP300*, a hub chromatin regulator with several regulatory roles in diverse biological contexts, including human salivary gland physiology (Rettig et al. 2016) and a GRN marker that defines a venom gland cell subpopulation (supplementary fig. S6*C*, Supplementary Material online). Our analyses of regulon activity associated with venom gene expression further emphasize how venom GRNs have been directly linked to (i.e., co-opted from) other secretory cell signaling pathways. The correlated expression of several other housekeeping genes related to protein folding (e.g., *DNAJC3*, *HSP90B1*, *PDIA4*, and *PDIA6*) and chromatin remodeling (e.g., *CARM1*, *TADA2A*, and *PBRM1*) further highlights the coordinated role of suites of nonvenom genes in supporting venom gene expression (Barua and Mikheyev 2021; Zancolli et al. 2022) and the extreme physiological demands on venom secretory cells (supplementary fig. S7, Supplementary Material online). Together, our results contribute to a new appreciation for the degree to which venom gland physiology and venom regulation have been inherently evolutionarily intertwined, partially through the direct co-option of secretory cell regulatory pathways to directly regulate venom genes expression.

Our results provide new independent evidence from single-cell data for the expression of several ERK- and UPR-related TFs being predictive of venom gene expression, broadly supporting the roles of a suite of TFs previously proposed to have been co-opted to regulate venom (Perry et al. 2022). In addition to TFs in these pathways that directly regulate venom by binding venom gene CREs, we find evidence that other TFs in these pathways are likely higher-level regulators within the signaling cascade associated with variation in venom expression across cells. These findings also provide new evidence that venom loci have convergently recruited the same higher-level signaling programs to regulate distinct venom gene families and that this pattern of recruitment has major ramifications for the evolution of heterogeneous venom GRN activation—fundamentally coupling their expression with distinct phases of dynamic signaling responses inherent in secretory cells. Our findings imply that this pattern of TF co-option is nonrandom and instead effectively manifests in heterogeneous venom gene expression across secretory cells. This conclusion further begs the question of what ultimate factors or constraints may underlie the evolution of such heterogeneous expression programs.

Prior spatial and single-cell studies have posited that observed venom gland expression heterogeneity may be driven by selection to avoid molecular or cellular constraints but have not nominated specific factors underlying such constraints. We hypothesize that a potential constraint could be the tandemly duplicated nature of many venom gene families, where the proximity of tandemly duplicated paralogs might impose steric constraints on gene expression of adjacent paralogs due to an inherent lack of space for the binding of transcriptional machinery to DNA. Based on observed heterogeneity in the notably compact PLA_2_ array, our results suggest that such steric hindrance may indeed constrain the coexpression relationships of tandem paralogs separated by small intergenic distances (fig. 6*A*), analogous to constraints identified on the coexpression of overlapping or tandem genes (Meyer and Beslon 2014; Zafar et al. 2014).

Intergenic distance, however, does not explain correlations within the much larger SVMP or SVSP arrays. The PLA_2_ array is hypothesized to be contained within a single *CTCF* loop, with further independent *CTCF* binding sites between each paralog (Perry et al. 2022), and tends to show discrete paralog expression across distinct cell subpopulations consistent with a role of chromatin structure in heterogeneity (fig. 6*B*). The much larger SVMP and SVSP arrays are both hypothesized to form more complex loop structures and contain multiple predicted *CTCF* binding sites (Perry et al. 2022) (fig. 6*C* and *D*), though these characteristics generally do not correspond strongly with observed patterns of heterogeneity. Separately, evidence for the expression of PLA_2_ inhibitor peptides in distinct subpopulations of venom secretory cells suggests that negative protein–protein interactions may also impose constraints that ultimately drive cellular heterogeneity to some extent. In summary, while intergenic distances, bound insulators, and protein–protein interactions may explain some proximal mechanisms or constraints that favor cellular heterogeneity, they fail to explain the underlying regulatory mechanisms that generate extensive venom gene expression heterogeneity across secretory cells that enable extreme expression of several multigene families in a single tissue.

Venom systems provide a unique perspective on the broad strategies and constraints that may govern the evolution of novel traits through the rewiring of GRNs. Our findings suggest that there may be more constraints on the evolution of GRNs than previously appreciated—including having to comply with constraints imposed by cellular physiology and chromatin. Our inferences highlight an elegant evolutionary solution to this problem in snake venom, where new GRN components appear to have been nonrandomly co-opted from an established highly dynamic set of regulatory cascades (ERK and UPR) that exhibit phased responses to glandular depletion. This strategy effectively generates expression heterogeneity within and among venom gene families, suggesting that similar constraints and analogous evolutionary solutions may be relevant for the rewiring of GRNs in other multigene families expressed in the same tissue.

Recent work to understand the evolutionary causes and consequences of snake venom composition and diversity has suggested that the expansion of venom gene families (e.g., increasing the numbers of paralagous copies) may be driven by selection to increase venom expression, by effectively increasing the dosage of genes (Margres et al. 2017a, 2017b). Other recent studies have identified evidence of strong balancing selection on snake venom loci consistent with predator–prey coevolution likely in response to prey resistance (Schield et al. 2022), indicating that multiple diverged paralogs may be beneficial for circumventing prey resistance. Under either one of these hypotheses, evidence presented here indicates that expression of multiple venom toxins requires more than simple gene duplication, and expression of many distinct toxins (paralogs or distinct gene families) may require the evolution of staged heterogeneous gene expression networks.

Over 100 animal lineages have evolved venom systems (Schendel et al. 2019), and there is emerging evidence that ERK and UPR are also involved in venom secretory systems in other animals (Barua and Mikheyev 2021; Zancolli et al. 2022). This suggests that the inherently heterogeneous properties of ERK and/or UPR pathways may have also been convergently co-opted for the regulation of multiple distinct venom systems. Collectively, this and prior studies suggest that these two conserved pathways may be particularly relevant raw material for co-option by new GRNs in which extremely high expression of multiple gene families in the same tissue is favored by selection. Several studies in venomous invertebrates have identified evidence of spatially and/or temporally heterogeneous patterns of venom production (Dutertre et al. 2014; Columbus-Shenkar et al. 2018; Walker et al. 2018; Verdes et al. 2022), including cellular variation in venom expression (Sachkova et al. 2019; Steger et al. 2022), suggesting that such heterogeneity may be a common theme in venom secretory tissues even across highly divergent animal lineages. It remains an open question, however, whether the molecular mechanisms underlying these broad patterns of cellular heterogeneity in other venomous lineages have arisen independently or if they are in some way linked to the activity of underlying GRNs as we describe here for snake venom systems. Future comparative studies across diverse venomous lineages that leverage single-cell multiome data (i.e., per-cell ATACseq coupled with RNA sequencing [RNAseq]) would be valuable for testing the generality of such evolutionary strategies for regulating venom and for understanding the proximate and ultimate causes and consequences of cellular heterogeneity.

## Methods

### Tissue Sampling and snRNAseq

An adult prairie rattlesnake (*C. viridis*) was collected from Weld County, Colorado under permits from Colorado Fish and Game (21HP0974 to S.P. Mackessy) and then housed and sampled at the University of Northern Colorado under approved and registered IACUC protocol 2004D-SM-S-23 (S.P. Mackessy). We manually extracted venom 1 day before humane sacrifice via deep anesthesia with isoflurane followed by dissection. Venom gland tissue was removed and snap frozen in liquid nitrogen. Frozen venom gland tissue was sent to SingulOmics Corporation (Bronx, New York) for single-nucleus isolation according to their standard protocol (McAlpin et al. 2022) and sequencing using the 10× Genomics Chromium system with Next GEM 3′ Single Cell Reagent kits v3.1 (10 × Genomics, Pleasanton, CA, USA). In brief, this protocol includes tissue homogenization and lysing to isolate nuclei followed by purification, resuspension, and dilution of the sample for 10× capture and library preparation. This 10× Genomics Chromium single-nucleus library was then sequenced on a single lane of an S4 flow cell on an Illumina NovaSeq 6000.

### Tissue-Level RNAseq and Analysis

We used a Trizol reagent protocol (Life Technologies, Carlsbad, CA, USA, No. 15596–026) to extract total RNA from the snap-frozen venom gland that was sent to Novogene Co. (Davis, CA, USA) for library preparation and sequencing. Poly-A-selected mRNA libraries were constructed with the TruSeq Stranded mRNA kit (Illumina) and sequenced on an Illumina NovaSeq to produce 150-bp paired-end reads. Raw reads were trimmed with default settings in Trimmomatic v0.39 (Bolger et al. 2014) and mapped to the annotated *C. viridis* genome with STAR v2.7.10a (Dobin et al. 2013). For this study, the reference genome annotation (Schield et al. 2019) was augmented with additional annotated SVSP genes (Perry et al. 2022) and myotoxin (Gopalan et al. 2022), which were venom genes not included in the originally published reference annotation. Similarly, because the reference genome did not include the mitochondrial genome sequence, the mitochondrial genome was included to allow the exclusion of cells with excess expression of mitochondrial genes from the study (Ilicic et al. 2016). We estimated gene expression counts using featureCounts v1.6.3 (Liao et al. 2014). Additionally, we remapped existing *C. viridis* multitissue RNAseq (Schield et al. 2019) to the augmented reference for comparison of venom gene expression. Counts were imported and normalized with DESeq2 v1.30.12 in R (Love et al. 2014). We incorporated the snRNAseq as pseudobulk by summing expression across all cells.

### snRNAseq Cell Identification and Analysis of Heterogeneity

Reads for snRNAseq were mapped to the augmented reference *C. viridis* genome with the 10× Genomics Cell Ranger 6.0.1 pipeline (Zheng et al. 2017) and imported to R for downstream analysis with Seurat v4.1.1(Hao et al. 2021), including normalization, filtering, clustering and subclustering, marker identification, dimension reduction, expression scaling, and plots of feature expression. Venom gene family enrichment was measured using custom gene lists in the escape v.1.6.0 R package (Borcherding and Andrews 2022), using the “enrichIt” function with default parameters. We tested for gene coexpression across cells using the scran v1.24.0 R package (Lun et al. 2016), with emphasis on venom genes, TFs previously associated with venom (Perry et al. 2022), and newly identified nonvenom gene markers.

We repeated the principal component analysis (PCA) and naïve clustering using the following venom gene families with moderate to high expression in *C. viridis*: SVMPs, SVSPs, myotoxin, PLA_2_s, bradykinin-potentiating protein (BPP), L-amino acid oxidases (LAAO), ohanin, cysteine-rich secretory proteins (CRISPs), and C-type lectins (CTLs). Predictions of venom gene region structures were used from a previous study (Perry et al. 2022).

### GRN Analyses

SCENIC v1.1.2 (Aibar et al. 2017) was used to identify major TFs and coregulated suites of direct interactors (regulons) from single-cell expression data. The workflow identified a total of 96 regulons that were manually annotated into four groups from literature sources (supplementary table S1, Supplementary Material online). The raw RNA counts matrix output by the Cell Ranger pipeline from a previous step was used as input to the program per the author's recommendation, and *C. viridis* gene names were converted to human ortholog names using an orthology table (Perry et al. 2020). The matrix was filtered with default parameters, which left 3,620 genes matched in the RcisTarget database (Aibar et al. 2017). Human TF motif databases scored using seven orthologous species were downloaded from https://resources.aertslab.org/cistarget/ and were used in the SCENIC workflow. We annotated factors as “chromatin modifiers” if, according to the literature, they either directly facilitate looping (e.g., *CTCF* and *ZNF143*), histone modification (e.g., *HDAC2*, *KDM5A*, *KDM5B*, and *RBBP5*), or tether or otherwise interact with chromatin-modifying complexes (e.g., *MGA*, *RUNX1*, and *SIN3A*). This conservatively excludes several factors that bind DNA but have no known role in directly remodeling chromatin.

The regulon activity–venom gene expression correlations were produced using the *rcorr* function from the Hmisc v4.7.1 package in R. Lowly correlated regulons were trimmed from the matrix by retaining those with average gene-wise correlations above that of *FOXP4*. This was done because it had the effect of retaining a single dendrogram cluster with other highly correlated regulons compared with first-pass clustering.

### Analyses of ERK and UPR

ERK and UPR gene set enrichment were measured as described above using custom gene lists and used for the correlation analyses. We used an elbow plot to determine an appropriate number of clusters and then used k-means clustering (*k* = 3) to cluster cells along the ERK–UPR enrichment regression. ATACseq footprint scores for TFs binding to venom gene CREs (promoters and enhancers) were obtained from a prior study (Perry et al. 2022). TF–gene network adjacency weights were calculated using the random forest regression algorithm GENIE3 (Huynh-Thu et al. 2010) in R. Of the 161 candidate venom regulating TFs identified based on differential expression and superenhancer association data (Perry et al. 2022), 148 were expressed in our sample. These TFs were used as “regulators” and all venom genes as “targets” in the call to the main GENIE3 function. The GENIE3 function “getLinkList” was used to identify top TF predictors of each venom gene.

## Supplementary Material

evad109_Supplementary_Data

## Data Availability

The venom gland snRNAseq data generated in this study has been submitted to the NCBI under BioProject accession PRJNA912694. Codes for reproducing analyses and figures are available at https://github.com/akwestfall/VenomSingleNucleus. Additional information required to reanalyze the data reported here is available upon request to the lead contact.
