# Supplementary material for: Single-Cell Heterogeneity in Snake Venom Expression Is Hardwired by Co-Option of Regulators from Progressively Activated Pathways
Source: Genome Biol Evol. 2023 Jun 13;15(6):evad109. doi: 10.1093/gbe/evad109 (PMC10289209; doi:10.1093/gbe/evad109)
Supplement: evad109_Supplementary_Data [file evad109_supplementary_data.zip › VenomGland_scRNA_SI_2023.05.16.pdf]

Supplementary Information for

Single-cell heterogeneity in snake venom expression is hardwired by co-option of regulators from progressively activated pathways

Aundrea K. Westfall[1*], Siddharth S. Gopalan[1*], Blair W. Perry[1,2], Richard H. Adams[3], Anthony Saviola[4], Stephen P. Mackessy[5], and Todd A. Castoe[1, §]

[1]Department of Biology, 501 S. Nedderman Dr., The University of Texas Arlington, Arlington, TX 76019 USA

[2]Department of Biology, Washington State University, Pullman, WA 99164, USA

[3]Department of Biological and Environmental Sciences, Georgia College and State University, Milledgeville, GA, USA

[4]Department of Biochemistry and Molecular Biology, 12801 East 17th Avenue, University of Colorado Denver, Aurora, CO 80045 USA

[5]School of Biological Sciences, University of Northern Colorado, Greeley, CO 80639, USA

[*]joint first authors; these authors contributed equally

[§]**Correspondence**: Todd A. Castoe, Department of Biology, University of Texas at Arlington, Arlington, TX 76010 USA

Email: todd.castoe@uta.edu

This PDF file includes:

Figures S1 to S9

SI References

Supplementary Figures

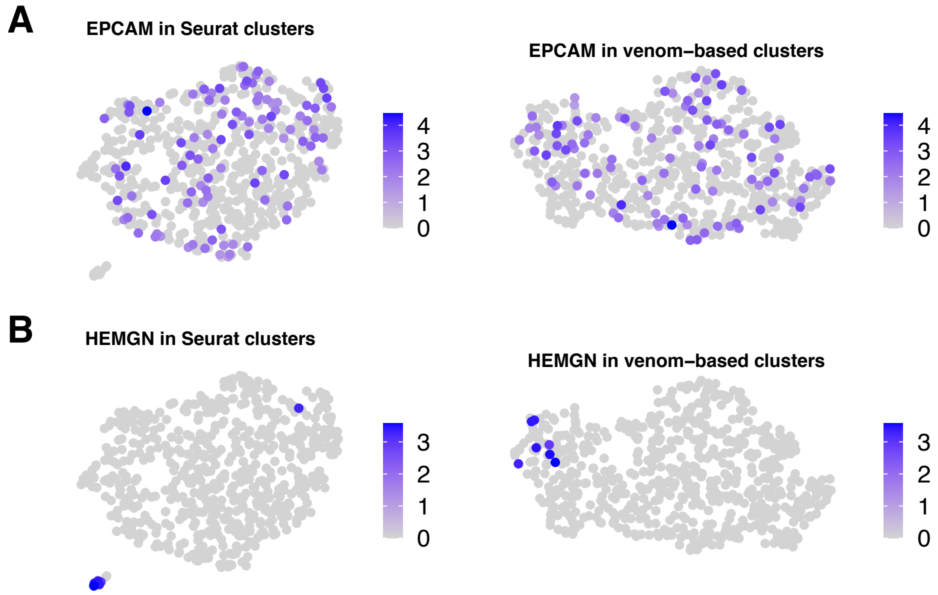

Fig. S1: Distribution of EPCAM (A) and HEMGN (B) as broad markers for epithelial cells and hematopoietic cells respectively.

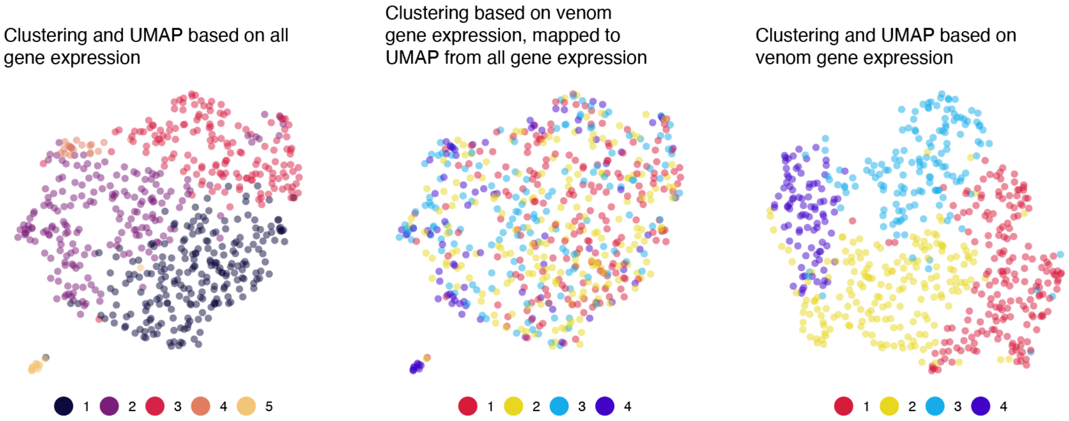

Fig. S2: Comparison of cluster identity under different gene sets. (A) Cluster identities determined by Seurat unsupervised clustering using algorithmically determined markers. (B) Cells mapped along UMAP from algorithmically determined markers but colored according to clusters determined by defined venom genes. (C) UMAP and clusters as determined by venom gene expression.

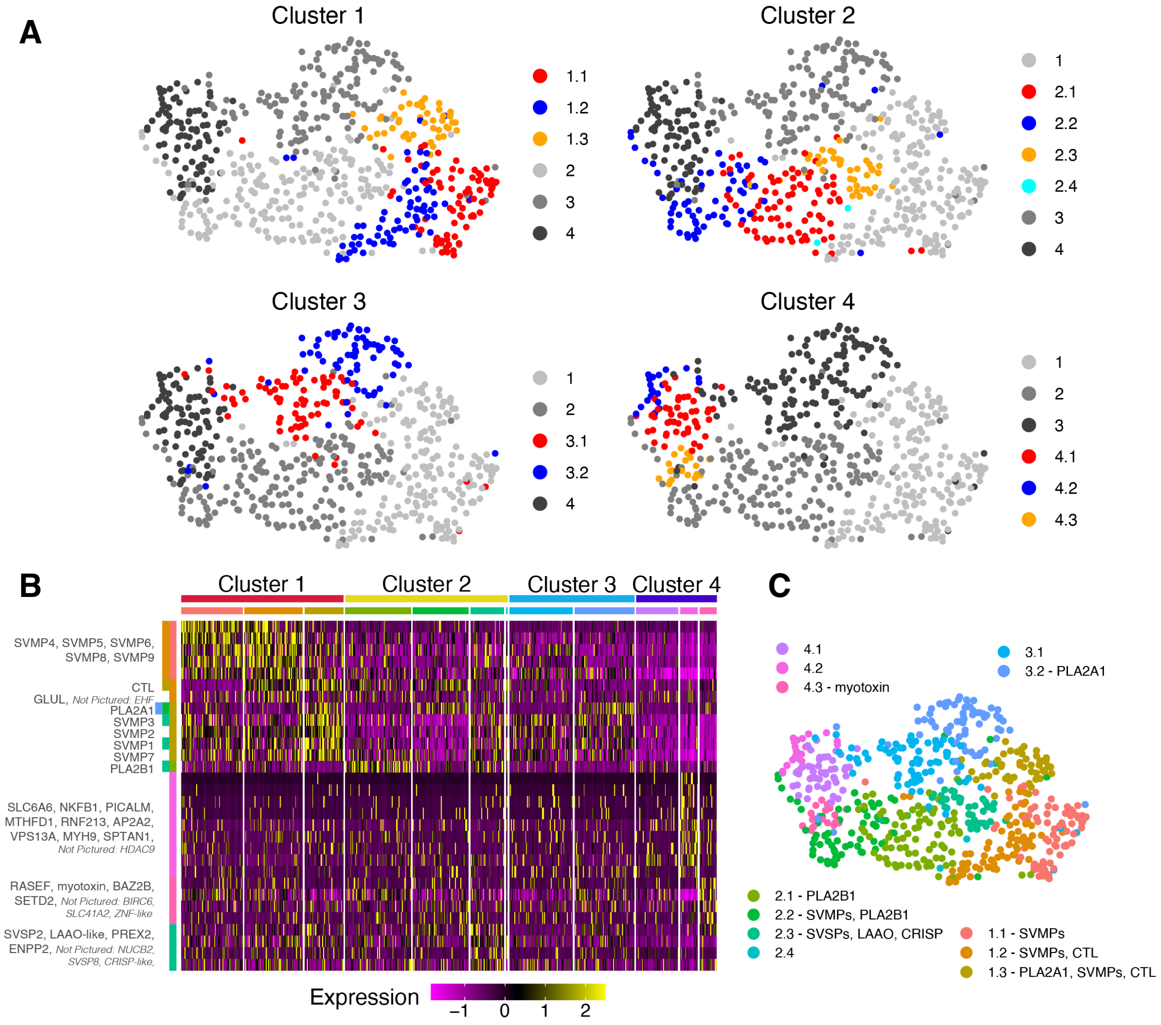

Figure S3: Sub-clustering of venom-based cell clusters reveals further venom gene heterogeneity. (A) Subcluster identity in each of four venom gene-based cell clusters. (B) Scaled expression of top markers for each subcluster, some shared by multiple subclusters. Some genes did not pass filtering for variance stabilizing transformation as part of visualization. (C) Visualization of all subclusters, listed with subcluster-specific enrichment for venom genes or gene groups.

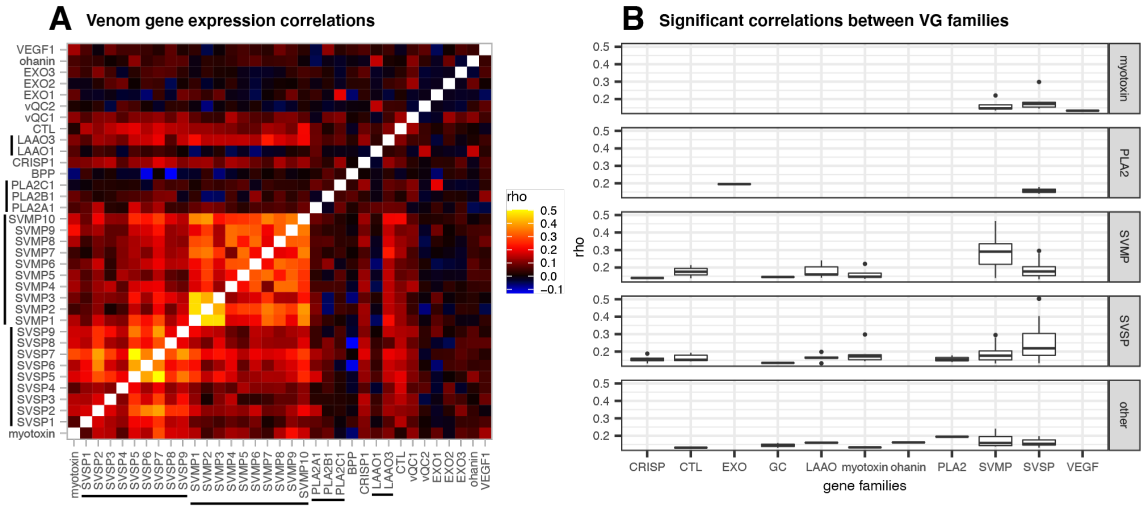

Figure S4: Correlation of venom gene expression within cells. (A) Heatmap of correlation coefficients for expression of venom genes. Lines indicate major venom gene families. (B) Significant correlations (FDR ≤ 0.05) between venom genes organized by gene family.

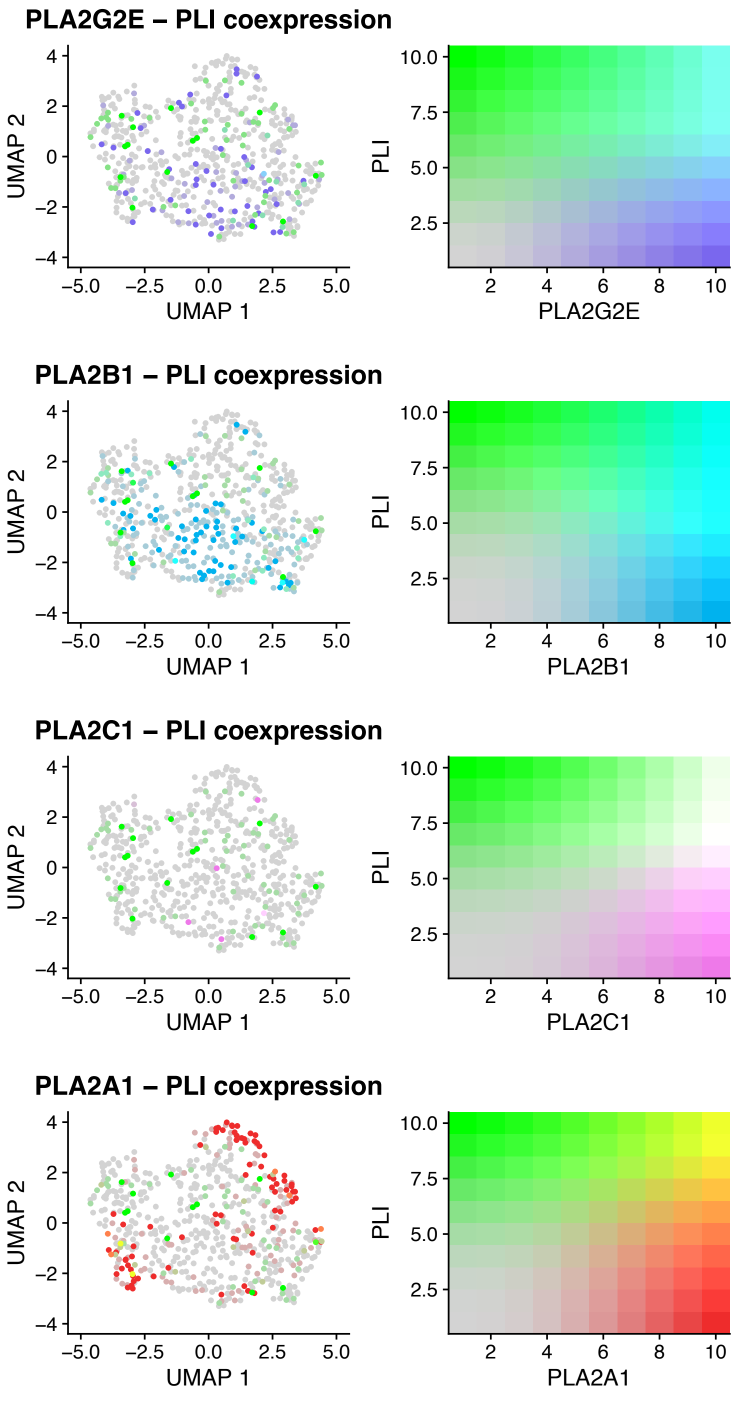

Figure S5: PLA2 and PLI coexpression plots. Coexpression of PLA2 genes with PLA2 inhibitor (PLI). Double negative cells are shown in grey.

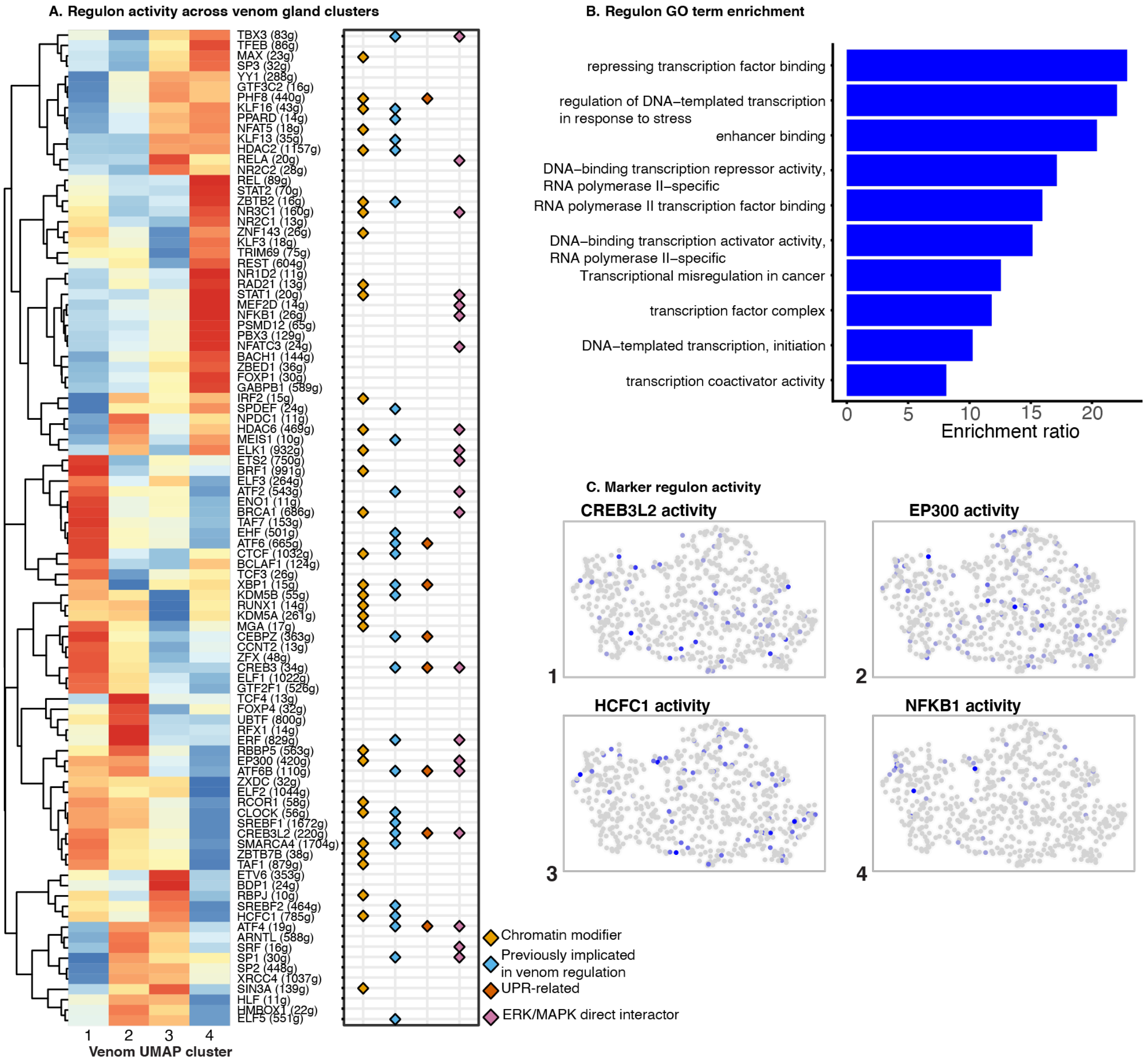

Figure S6: Analysis of the regulons identified by SCENIC. (A) Heatmap of regulon activity scores (blue=low, red=high) measured by AUCell (Aibar et al., 2017) across the 4 venom UMAP clusters. Number of genes in each regulon is shown in parentheses. Manual categorization of regulons into 4 categories is shown in the box to the right. (B) GO term and KEGG pathway enrichment of the 96 regulons identifies broad role of regulons in transcription activity. (C) Activity for cluster-specific regulons identified using regulon specificity scores calculated following the method from Suo et al. (2018) in SCENIC.

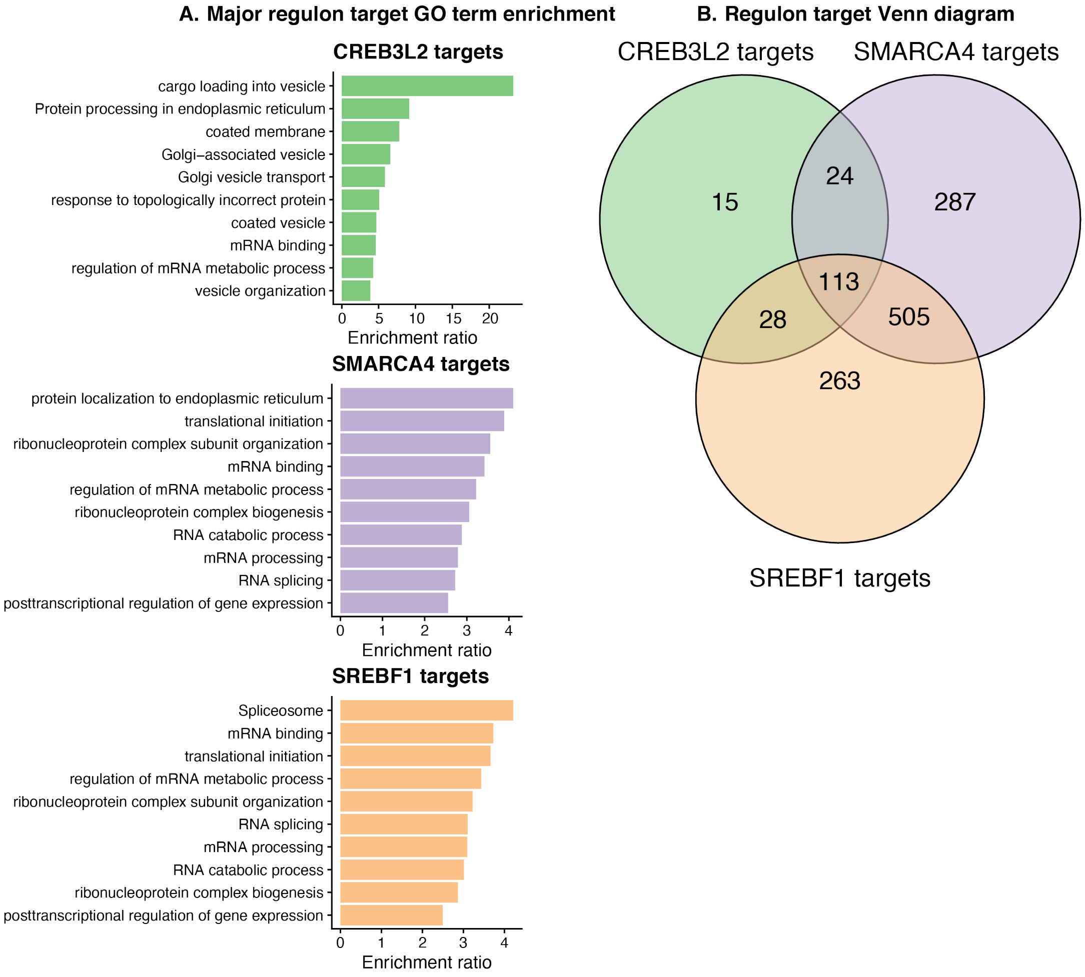

Figure S7: Venom expression correlated regulons share genes and function. (A) GO term and KEGG pathway enrichment of the filtered transcription factor targets of the three most correlated regulons, including targets shared between regulons. (B) Venn diagram of target genes in regulons illustrates shared transcription factors.

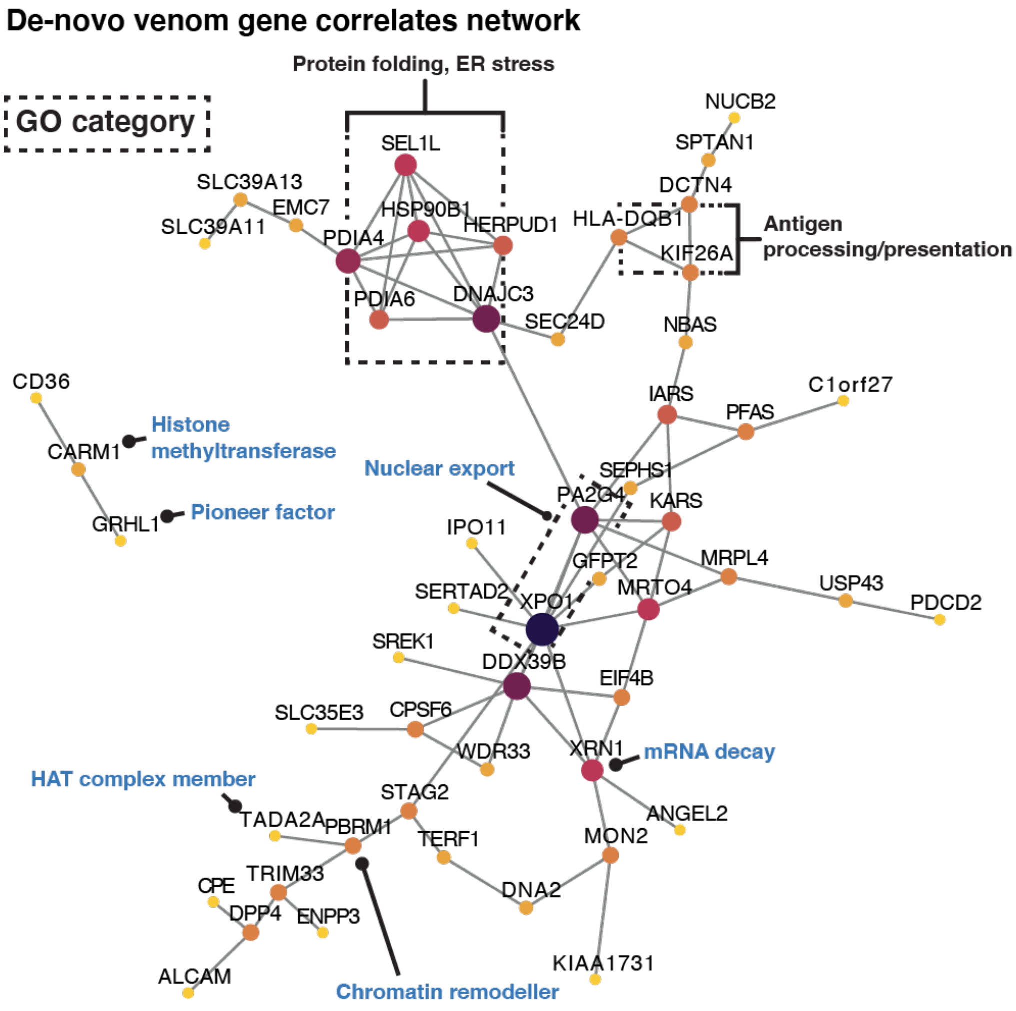

Figure S8: Naïve network formed using lower-level non-venom genes. Significantly venom-associated non-venom genes illustrates a set of genes enriched for GO terms related to protein misfolding, and mRNA export and degradation, and antigen presentation. Other genes (in blue) are known chromatin modifiers and pioneer transcription factors (node color and size represent degree).

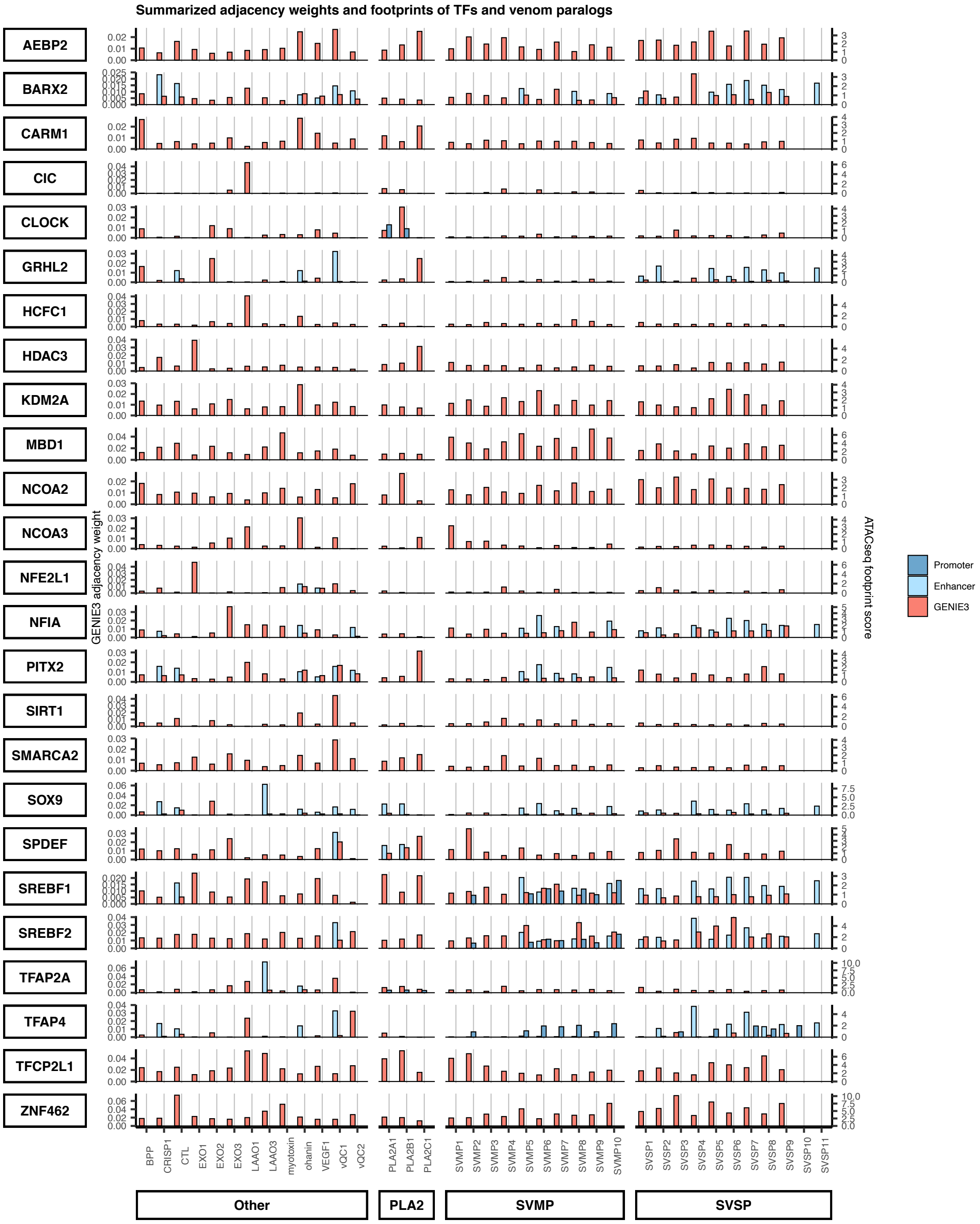

Figure S9: Additional transcription factors outside of ERK and UPR signaling contribute to venom gene regulation.

SI References

Al-Furoukh N, Ianni A, Nolte H, Hölper S, Krüger M, Wanrooij S, Braun T. 2015. ClpX stimulates the mitochondrial unfolded protein response (UPRmt) in mammalian cells. *Biochimica et Biophysica Acta (BBA) - Molecular Cell Research* 1853:2580–2591.

Bailey SD, Zhang X, Desai K, Aid M, Corradin O, Cowper-Sallari R, Akhtar-Zaidi B, Scacheri PC, Haibe-Kains B, Lupien M. 2015. ZNF143 provides sequence specificity to secure chromatin interactions at gene promoters. *Nature Communications 2015 6:1* 6:1–10.

Blackledge NP, Klose RJ. 2021. The molecular principles of gene regulation by Polycomb repressive complexes. *Nature Reviews Molecular Cell Biology 2021 22:12* 22:815–833.

Christova R, Jones T, Wu PJ, Bolzer A, Costa-Pereira AP, Watling D, Kerr IM, Sheer D. 2007. P-STAT1 mediates higher-order chromatin remodelling of the human MHC in response to IFNγ. *J Cell Sci* 120:3262–3270.

Daftary GS, Lomberk GA, Buttar NS, Allen TW, Grzenda A, Zhang J, Zheng Y, Mathison AJ, Gada RP, Calvo E, et al. 2012. Detailed structural-functional analysis of the Krüppel-like factor 16 (KLF16) transcription factor reveals novel mechanisms for silencing Sp/KLF sites involved in metabolism and endocrinology. *Journal of Biological Chemistry* 287:7010–7025.

Doi M, Hirayama J, Sassone-Corsi P. 2006. Circadian Regulator CLOCK Is a Histone Acetyltransferase. *Cell* 125:497–508.

Fang Y, Yan J, Ding L, Liu Y, Zhu J, Huang C, Zhao H, Lu Q, Zhang X, Yang X, et al. 2004. XBP-1 increases ERα transcriptional activity through regulation of large-scale chromatin unfolding. *Biochem Biophys Res Commun* 323:269–274.

Gatta E, Grayson DR, Auta J, Saudagar V, Dong E, Chen Y, Krishnan HR, Drnevich J, Pandey SC, Guidotti A. 2019. Genome-wide methylation in alcohol use disorder subjects: implications for an epigenetic regulation of the cortico-limbic glucocorticoid receptors (NR3C1). *Molecular Psychiatry 2019 26:3* 26:1029–1041.

Giaimo BD, Ferrante F, Vallejo DM, Hein K, Gutierrez-Perez I, Nist A, Stiewe T, Mittler G, Herold S, Zimmermann T, et al. 2018. Histone variant H2A.Z deposition and acetylation directs the canonical Notch signaling response. *Nucleic Acids Res* 46:8197–8215.

Gong F, Clouaire T, Aguirrebengoa M, Legube G, Miller KM. 2017. Histone demethylase KDM5A regulates the ZMYND8–NuRD chromatin remodeler to promote DNA repair. *Journal of Cell Biology* 216:1959–1974.

Huang C, Sloan EA, Boerkoel CF. 2003. Chromatin remodeling and human disease. *Curr Opin Genet Dev* 13:246–252.

Klein BJ, Piao L, Xi Y, Rincon-Arano H, Rothbart SB, Peng D, Wen H, Larson C, Zhang X, Zheng X, et al. 2014. The histone-H3K4-specific demethylase KDM5B Binds to its substrate and product through distinct PHD fingers. *Cell Rep* 6:325–335.

Kwon CS, Wagner D. 2007. Unwinding chromatin for development and growth: a few genes at a time. *Trends in Genetics* 23:403–412.

Li QJ, Yang SH, Maeda Y, Sladek FM, Sharrocks AD, Martins-Green M. 2003. MAP kinase phosphorylation-dependent activation of Elk-1 leads to activation of the co-activator p300. *EMBO J* 22:281–291.

Liao Y, Wang J, Jaehnig EJ, Shi Z, Zhang B. 2019. WebGestalt 2019: gene set analysis toolkit with revamped UIs and APIs. *Nucleic Acids Res* 47:W199–W205.

Lichtinger M, Hoogenkamp M, Krysinska H, Ingram R, Bonifer C. 2010. Chromatin regulation by RUNX1. *Blood Cells Mol Dis* 44:287–290.

Lin J, Zhao Y, Ferraro AR, Yang E, Lewis ZA, Lin X. 2019. Transcription factor Znf2 coordinates with the chromatin remodeling SWI/SNF complex to regulate cryptococcal cellular differentiation. *Communications Biology 2019 2:1* 2:1–14.

Lunazzi G, Buxadé M, Riera-Borrull M, Higuera L, Bonnin S, Huerga Encabo H, Gaggero S, Reyes-Garau D, Company C, Cozzuto L, et al. 2021. NFAT5 Amplifies Antipathogen Responses by Enhancing Chromatin Accessibility, H3K27 Demethylation, and Transcription Factor Recruitment. *The Journal of Immunology* 206:2652–2667.

Merkwirth C, Jovaisaite V, Durieux J, Matilainen O, Jordan SD, Quiros PM, Steffen KK, Williams EG, Mouchiroud L, Tronnes SU, et al. 2016. Two Conserved Histone Demethylases Regulate Mitochondrial Stress-Induced Longevity. *Cell* 165:1209–1223.

Mishiro T, Ishihara K, Hino S, Tsutsumi S, Aburatani H, Shirahige K, Kinoshita Y, Nakao M. 2009. Architectural roles of multiple chromatin insulators at the human apolipoprotein gene cluster. *EMBO J* 28:1234–1245.

Mullan PB, Quinn JE, Harkin DP. 2006. The role of BRCA1 in transcriptional regulation and cell cycle control. *Oncogene 2006 25:43* 25:5854–5863.

Odho Z, Southall SM, Wilson JR. 2010. Characterization of a novel WDR5-binding site that recruits RbBP5 through a conserved motif to enhance methylation of histone H3 lysine 4 by mixed lineage leukemia protein-1. *Journal of Biological Chemistry* 285:32967–32976.

Olivieri D, Paramanathan S, Bardet AF, Hess D, Smallwood SA, Elling U, Betschinger J. 2021. The BTB-domain transcription factor ZBTB2 recruits chromatin remodelers and a histone chaperone during the exit from pluripotency. *Journal of Biological Chemistry* 297.

Peng Haoyang, Zhang Simin, Peng Yihan, Zhu Shuangyi, Zhao Xin, Zhao Xiaocong, Yang Shuangshuang, Liu Guangxue, Dong Yang, Gan Xiaoli, et al. 2021. Yeast Bromodomain Factor 1 and Its Human Homolog TAF1 Play Conserved Roles in Promoting Homologous Recombination. *Advanced Science* 8:2100753.

Perry BW, Gopalan SS, Pasquesi GIM, Schield DR, Westfall AK, Smith CF, Koludarov I, Chippindale PT, Pellegrino MW, Chuong EB, et al. 2022. Snake venom gene expression is coordinated by novel regulatory architecture and the integration of multiple co-opted vertebrate pathways. *Genome Res* 32:1058–1073.

Ren G, Cui K, Zhang Z, Zhao K. 2015. Division of labor between IRF1 and IRF2 in regulating different stages of transcriptional activation in cellular antiviral activities. *Cell Biosci* 5:1–10.

Rivera C, Lee HG, Lappala A, Wang D, Noches V, Olivares-Costa M, Sjöberg-Herrera M, Lee JT, Andrés ME. 2022. Unveiling RCOR1 as a rheostat at transcriptionally permissive chromatin. *Nature Communications 2022 13:1* 13:1–15.

Yu S, Zhou C, Cao S, He J, Cai B, Wu K, Qin Y, Huang X, Xiao L, Ye J, et al. 2020. BMP4 resets mouse epiblast stem cells to naive pluripotency through ZBTB7A/B-mediated chromatin remodelling. *Nat Cell Biol* 22:651–662.
